# Nursing students’ experiences of service-learning at community and hospital pharmacies in Belize: Pedagogical implications for nursing pharmacology

**DOI:** 10.1371/journal.pone.0276656

**Published:** 2022-11-03

**Authors:** Danladi Chiroma Husaini, David D. Mphuthi, Jane A. Chiroma, Yusuf Abubakar, Adeniyi O. Adeleye

**Affiliations:** 1 Faculty of Health Sciences, Allied Health Department, University of Belize, Central America, Belmopan, Belize; 2 Faculty of Health Studies, College of Human Sciences, University of South Africa, Pretoria, South Africa; 3 Department of Leadership and Curriculum Development, Pan Africa Christian University, Nairobi, Kenya; 4 Central Queensland University, College of Nursing and Midwifery, Rockhampton, Queensland, Australia; Kent State University, UNITED STATES

## Abstract

**Objectives:**

Many students seem to find pharmacology learning very challenging due to the complexity and variety of drugs they have to study. The number of drugs the students have to learn, the duration of time to learn the medications, and the evolving nature of diseases demanded learning beyond the classroom walls. This study explored and described nursing students’ experiences in community and hospital-based pharmacy practice sites during their service-learning and its implications for pharmacology pedagogical practices.

**Methods:**

Kolb’s learning theory provided the framework to explore nursing students’ 48-hour service-learning experiences at community/hospital-based pharmacies in Belize and its implications for pharmacology pedagogy. The study utilized two qualitative approaches, reflective journals and focus group interviews, to collect data from 46 second-year nursing students. NVivo software and coding schemes were employed to analyze the data from the interviews and reflective journals.

**Results:**

Students reported learning medications, integrating classroom pharmacological knowledge at pharmacy practice sites, acquiring and enhancing communication skills, interpreting prescriptions, dispensing medications, drug calculations, taking inventory, doing vital signs, and patient education. In addition, students reported experiencing inter-professional relationships as healthcare team members. Anxiety was a major challenge experienced by many students at the beginning of the service-learning experience.

**Conclusions:**

This study highlights the importance of experiential learning of pharmacology amongst second year nursing students, offering the opportunity to inform and support pharmacotherapeutics educators in designing strategies for more effective teaching of medications to nursing students. It also supports the addition of pharmacy placements to the nursing curriculum’ as it shows that nursing students can learn medications, skills, and teamwork from experiential pharmacy site posting. Combining classroom instruction with pharmacy experiential service learning might be an effective complement for teaching nursing pharmacology.

## Introduction

Pharmacotherapeutics is the study of medications and their clinical use in disease management. It comprises pharmacokinetics, pharmacodynamics, and therapeutics. In nursing practice, pharmacotherapeutics is a crucial component of drug administration that embraces assessment, planning, implementation, and evaluation. The nurse’s role involves prescription interpretation, dosage calculation, drug administration, and monitoring of the effects of the administered drug [1]. Many students find pharmacology difficult because of the complexity of drugs, the number of drugs the students have to learn, the duration of time to learn the drugs, and the evolving nature of diseases the students are required to understand [2]. Few studies have reported the dissatisfaction of nursing students with pharmacology teaching in their training, resulting in anxiety and uncertainty in medication management and decision-making [3,4]. Another concern for pharmacotherapeutics teaching and knowledge development is the minimization or elimination of medication errors during practice [2]. Even though other healthcare professionals can cause medication errors, nursing medication errors have been the most common because nurses administer most drug orders and spend a considerable amount of time with their patients [5]. Medication errors can occur during the preparation, distribution, or administration of drugs. Research has shown that approximately one-third of medication errors occur during the nurse administration phase and contribute to the adverse effects that compromise patient safety [6]. Medication errors have been reported to prolong patient hospitalization, leading to increased financial costs and therapy burden [5,6].

Some pharmacology teaching methods and strategies have been described to undergraduate students. Such strategies include traditional lectures, simulation, autobiography of drugs, concept maps, flipped classrooms, collage making, animations, cooperative learning, and integrating theory with clinical exposure [2,7–9]. Traditional nursing classroom lectures are mainly effective for cognitive content delivery and do not provide sufficient experiential learning [10]. With the expanding scope of diseases and the number and complexity of medications, the need to urgently explore innovative strategies in enhancing both the cognitive and experiential learning in nursing pharmacology is paramount. This will ensure effective theoretical, cognitive and experiential learning opportunities for enhance holistic nursing training that leads to high quality and safe patient care.

Service-learning, as the experiential learning component, is an innovative teaching method designed to integrate educational objectives with community needs to enhance students’ learning during community participation [11–14]. Furthermore, service-learning connects students to the realities of practice and comprises reciprocal learning, reflection, and experiential learning as essential elements that distinguish service-learning from other learning experiences [11,13].

Currently, few studies have explored and described nursing education in Central America and the Caribbean, to which Belize is a member [15–18]. Formal nursing education in Belize was established in 1894, approximately 21 years after the establishment of the first nursing schools in the United States of America [15]. Since then, the training of nurses in Belize has transitioned from awarding diplomas to baccalaureate degree training.

To provide students with a holistic model of care learning experience, we explored innovative service-learning teaching strategies to help students gain sufficient interest and knowledge in pharmacology [13,19,20]. This study’s pedagogical approach allowed nursing students to spend time at community and hospital pharmacies with licensed pharmacists to facilitate enhanced learning of pharmacology. The purpose of this study is to introduce second-year baccalaureate nursing students to experiential pharmacotherapeutics. To the best of our knowledge, this is the first study in Belize, Central America, and the Caribbean.

### Theoretical foundation

**Fig 1:**
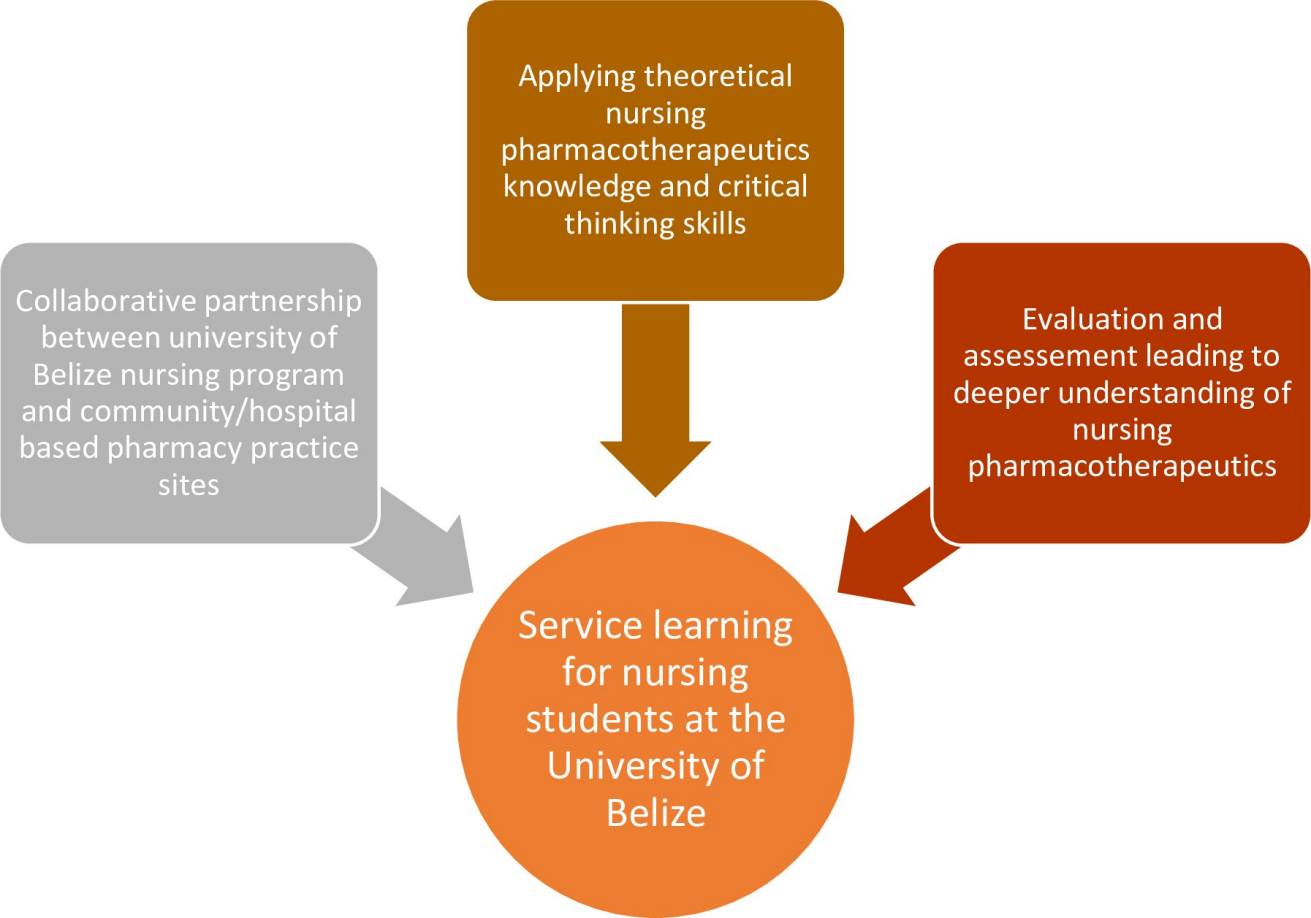
Service-learning framework.

The experiential learning theory developed by Kolb and Kolb [21] was adopted in this study. For over three decades, Kolb’s model of experiential learning has been used by educators to support pedagogical practices [21]. Concrete experience, reflective observation, abstract conceptualization, and active experimentation formed the key phases of Kolb’s experiential learning cycle [21]. The nursing undergraduate learner can start the experience (cognitive and/or experiential) at any phase of the learning cycle, thereby giving the student the prospect of applying the knowledge learned to develop the skills and competencies necessary for practice (Fig 1). Experiential learning inspires student nurses to relate to theoretical content while learning the skills that will help them make meaningful contributions to global healthcare.

### Problem statement

The enormous content of the nursing pharmacotherapeutics at the university of Belize (UB) and the short duration of time allotted for teaching have resulted in information overload to the students, creating a lack of interest in learning and increased student anxiety due to the inability to adequately retain pharmacological knowledge. This study explored the experiences of student nurses at pharmacy practice sites as part of an innovative service-learning teaching strategy to help students gain meaningful knowledge in pharmacotherapeutics.

### Primary question

1. How do volunteer service-learning-related activities at community/hospital-based pharmacies help nursing students learn pharmacotherapeutics?

### Secondary questions

1. In what ways do students’ pharmacy service-learning experiences expand their knowledge of pharmacotherapeutics?
2. As a pedagogical approach, what role does service-learning in community/hospital-based pharmacies practice sites play in developing nursing practices that count in nursing pharmacotherapeutics undergraduate education?

## Methodology

### Method

Kolb’s learning theory provided the framework to explore nursing students’ service-learning experiences in community/hospital-based pharmacies in Belize and its implications for pharmacology pedagogy [22,23]. Two qualitative approaches, reflective journals and focus groups were used in this study.

### Sampling

Students enrolled in the nursing pharmacotherapeutics course and who provided written consent to participate were included in the study. Any student who did not provide consent was excluded from the study. Fifty students enrolled in the course that started in January 2020 and ended in the first week of May 2020. Forty-six students completed all aspects of the study.

### Setting

The students volunteered for a minimum of 48 hours of service-learning at approved pharmacy practice sites within Belize. The approved pharmacy practice sites are mostly sites used by the University of Belize’s pharmacy program for the training of pharmacy students. A letter of introduction containing the volunteer service-learning objectives was given to the students to present to the preceptor pharmacist.

### Instruments for data collection

Two qualitative data, reflective journals and focus groups were obtained in this study. Kolb’s [23] experiential cycle was utilized along with concepts garnered from the literature review to formulate questions that guided students’ reflections and focus group discussions. The coding schemes proposed by Kember et al. [24] were employed for evaluating students’ reflective journals.

### Data collection

**Fig 2:**
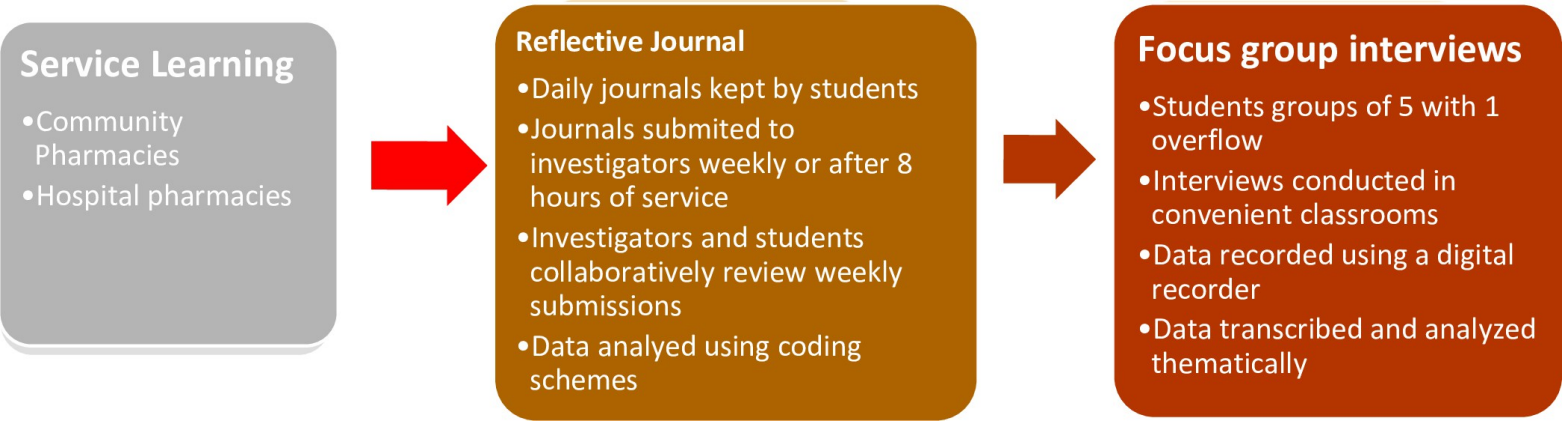
Approach for data collection and analysis.

Nursing students enrolled in the pharmacotherapeutics course were informed about the research protocols, and for the 46 students interested, consent was obtained. Qualitative data were collected from reflective journals and focus-group interviews (Fig 2).

#### Reflective journals

For the reflective journals, the students kept one paragraph of hand-written reflection daily for the duration of the experiential learning. The prompts for their daily journals were based on the service-learning objectives. Students submitted unnamed reflective journals weekly or at the end of every 8-hour service-learning duration. Approximately every student submitted six reflective journals throughout the service-learning period. Copies of the reflective journals were made for one of the reviewers. To enhance credibility of the study we reviewed the journal entries with students for clarity and to reduce the likelihood of investigators’ mistaken meanings in students’ writings. The discussion with the students helped the investigators better understand the students’ values, feelings, and thoughts expressed in the reflective journal entries. The reflective journal included the students’ experiences, the drugs they came across at the pharmacy, and information about the drug. The review allowed the investigators to guide the students and encouraged the reflective process while promoting pharmacologic learning.

#### Focus groups

For the focus group data collection, two instructors conducted the focus group interviews with student nurses. Because of the large class size, the 46 students were divided into nine groups of five students each with one overflow to facilitate robust discussions. The focus group interviews were conducted over a round table in comfortable classrooms at the University of Belize. Two of the study investigators conducted the focus group interviews using a semi-structured questionnaire. The main question asked was “*share with us your experience during the pharmacy volunteer service”* (S1 File). Thereafter, other follow-up questions were asked to understand how students experienced pharmacotherapy service-learning in community/hospital-based pharmacies. Each focus group session lasted for approximately one and half hours. Questions were presented to the students by the investigators, and responses were recorded based on group responses rather than on individual responses. Students’ responses were recorded using notes and digital recorders.

### Data analysis

#### Reflective journals

Two investigators analyzed the reflective journals to determine the student’s level of introspection based on whether the journal entry characterizes non-reflection, understanding, reflection, and critical thinking, as Kember et al. [24] coding schemes proposed. The approach was employed to identify students’ analytical and reflective insights and the ability to merge process, introspection, and analysis eventually.

#### Focus groups

For the data analysis, two investigators analyzed the focus group interviews. The audio-recorded interviews were first transcribed verbatim and analyzed thematically. The thematic analysis procedure summarized by Moseholm et al. [25] was employed as the general strategy for the focus groups. In order to obtain a sense of the whole, the interview transcripts were read several times. We then identified emergent themes, patterns, and uniqueness within the scripts. After that, similarities and differences within the narratives were compared to create coherent themes. Microsoft software was used to organize the data, while Nvivo 11 software aided the analysis. The coding of themes was based on group respondents’ views and levels of data saturation from both sources. The integrated themes and sub-themes from the focus group summarized the students’ service-learning experiences. Four main themes and seven subthemes were identified in the analysis. The main themes and sub-themes identified in the analysis are presented in Table 2 and discussed below. The consolidated criteria for reporting qualitative research, as reported by Tong et al. [26] and Holloway [27], guided the data analysis and reporting of our findings.

### Trustworthiness

Peer debriefing and member checks were employed to ensure trustworthiness during the qualitative data analysis. Peer debriefings facilitated an in-depth understanding of the students’ experiences within the pharmacy practice sites, and member checks were utilized to ensure credibility and accuracy of the results. In addition, transcripts and field notes were studied and discussed by the research team to ensure accurate interpretation and presentation of the data. Finally, the collaborative review of students’ unnamed reflective journals provided clarity and reduced the likelihood of investigators’ mistaken meanings in students’ writings, thereby strengthening the interpretation process.

### Ethics approval

This study was performed in accordance with the principles of the Declaration of Helsinki. The study was approved by the Health Sciences Faculty of the University of Belize as part of the faculty experiential learning project (UBP-ELP-1).

## Results

### Demographics

A total of 46 students comprised of male n = 11 (23.91%) and female n = 35 (76.09%) with a mean age of n = 22.06±5.76 participated in the study. The majority of the participants, n = 29 (63.04%), held a high school diploma, while n = 16 (34.78%) had associate degrees, with one (2.17%) rural health nurse. Approximately 83% (n = 38) volunteered at community pharmacies, 13% (n = 6) at hospital-based pharmacies, and 4% (n = 2) volunteered at community and hospital-based pharmacies. The mean hours of volunteer service reported were 50.43±5.37.

### Reflective journals

Most students expressed that collaborative discussions with the lecturers concerning the reflective journals enriched the service-learning experience. The collaborative reviews allowed students to discuss their experiences with the lecturers and receive timely feedback, helping the students gain insights, understanding, and better knowledge of medications. Many students indicated that the experience fostered a better relationship with the preceptors and the lecturers and encouraged pharmacologic learning.

The reflective journal analysis indicated that most students’ learning was enhanced as they reflectively journaled their daily experiences, as indicated by a student’s entry.

Today, I learned the mechanism of action, dosage, and dispensing information of few medications, notably omeprazole, lisinopril, and atorvastatin.

Overall, the analysis of the reflective journals showed a pattern based on the coding schemes described by Kember et al. [24]. The reflective journal entries made by most students were at the understanding and reflective levels; only a few provided entries that indicated critical reflection levels, as evidenced by a student’s reflective journal entry.

A patient came with a prescription to buy Ardosons. First, I was curious about the name because it was unusual and did not seem to follow a pattern, and when I checked the medication, I discovered it was a combination of indomethacin, betamethasone, and methocarbamol. I knew the mechanism of action of 2 of the meds, but I had not heard of the last one. After researching the third drug, I knew combining NSAIDs, corticosteroids, and muscle relaxants would help someone in pain. I then searched for the drug and discovered I was right. I shared the information of my discovery with some of my coursemates.

The students did not record many critical reviews in their reflective journals. Nevertheless, the course lecturers were satisfied with the student’s ability to demonstrate comprehension of basic pharmacologic knowledge through the reflective journals, achieving the intended learning objective.

### Focus groups

Themes and sub-themes identified during analysis of focus group transcripts (Table 1).

**Table 1:** Themes and sub-themes identified during analysis of focus group.

Focus Group Themes and Sub-themes
**1**. Student’s perceptions of pharmacy service-learning
**2.** Students integration of theoretical pharmacologic knowledge
**3.** Students acquisition of skills from pharmacy service learning **3.1** Pharmacy environment for learning drugs **3.2** Prescription interpretation **3.3** Calculating and dispensing medications **3.4** Taking drug inventory **3.5** Taking patients vital signs **3.6** Patient education **3.7** Communication skills in healthcare setting
**4.** Inter-professional relationship in healthcare
5. Challenges and suggestions to pharmacy service learning experience

### 1. Students’ perceptions of pharmacy service-learning

In this study, 46 students in focus group discussions shared their experiences and perceptions after a 48- hour pharmacy voluntary service indicated that the experience was interesting and helped them learn and remember medications differently. A student from group A indicated that the experience was satisfying, as expressed by the following quote:

*I knew that I had to learn medications to succeed in my career…*..*in the end*, *it turned out to be a great experience*. *I realized that I could learn from other medical professionals while serving*. *In the end*, *I benefitted more than the pharmacist because I achieved my goals of going there*. *In all*, *I had a very satisfying experience and would not mind volunteering again*. (Student 3 from Group A)

Some students reported that at first, they were not very enthusiastic about going to the pharmacy because in their opinions it was not a nursing setting and they were not sure how they will fit in a pharmacy setting. However, at the end of their volunteer service, they reported that they had learned several medications differently and in a different setting. Some students mentioned that they enjoyed their experiences as they were actively involved in learning and dispensing medications, communicating with clients, and attending to patients. Most of the students were of the view that this experience was beneficial and was an eye-opener to the demands of the nursing profession, especially when it comes to medications.

*I enjoyed my experience and felt I gained a lot of knowledge from it since*. *The experience helped me throughout my pharmacotherapy class…I really did enjoy the time of packing the medications*, *documenting*, *learning*, *and dispensing medication*. *All the knowledge gained at the pharmacy is now permanent and is helping me; it was worth the volunteer*. (Student 1 from Group B)

### 2. Students integration of theoretical pharmacogic knowledge

Students shared that volunteering at the pharmacy helped them to integrate the theoretical knowledge learned in class with the drugs encountered at the pharmacy. Pharmacologic concepts taught in the class were reinforced at the pharmacy practice sites. They reported that they had first-hand experiences with many medications in a way that was not taught in the class. Some students recounted that the volunteering initiative was very helpful in affirming what they learned in class. The students recounted that they learned the drug names, indications, mechanisms of action, side effects, and pregnancy categories. Students further indicated that the experience broadened their knowledge of medications and helped them learn. Integrating theory into practice is important for training nurses. The students described that the pharmacy experience helped them recall and remember some of the drugs taught in the classroom. They view that what they learned in class was reinforced in the pharmacy, or vice versa. The quotes from students are shown below:

Much medication information is provided during the class lecture, but when I go to the pharmacy, I see the drugs, read the information on the packaging, and sometimes dispense them. That helps me a lot with what was taught in the class and sometimes much more. (Student 4 from Group C).

"The combination of the class and the pharmacy volunteer allowed me to quickly grasp the information on medications, such as the mechanism of action, the drug side effects, uses, and even the pregnancy categories. I learned a lot." (Student 1 from Group I)

### 3. Student’s acquisition of skills from pharmacy service-learning

The majority of the students reported that they appreciated the volunteer experience because it helped them practically learn the medications. The students reported learning several skills that are useful for medication safety and nursing practice as summarized on Table 2. They also testified that they now understand part of their roles as members of the healthcare team. This helped to strengthen their previous knowledge and make learning memorable, as reported in the following quotes:

*The volunteer was more like a practical component of the pharmaco class as it blended so well with my learning methodology since I learned easily by seeing*. (Student 5 from Group E)

**Table 2:** Student’s acquisition of skills from pharmacy service-learning at community/hospital pharmacies in Belize.

Sub-themes	Comments	Student’s quotes
Pharmacy environment for learning drugs	Students appreciated pharmacy environment through cleaning shelves that provided the opportunity for learning medications and knowing how they are arranged.	*We were asked to clean cabinets and shelves in the prescription area of the pharmacy*. *While doing so*, *I had to remove the stacks of medications and cleaned them one by one*. *We also had to look for expiration dates of all the medications available in that section and had to tag the ones that would expire within six months*. *I got to know that the reason for doing this is for the pharmacist to dispense the ones that expire sooner than those that had a longer shelf life*. *Cleaning the shelves and arranging the medications was a special skill that I learned during the volunteer*. *Apart from cleaning*, *we were asked to help count medication tablets that the customers needed*. (Student 2 from group F)
Prescription interpretation	Students reported being taught to read and interpret prescriptions accurately so that the patient could be given the correct drug dosage.	*During class sessions*, *we were taught the rights of medication administration*. *I practically experienced that*, *as I learned to dispense medications to a few patients during the volunteer*. *I first had to learn to interpret the prescription correctly*, *find the medication*, *and come back to dispense to the patient*. *Interpretation of the prescription was a very important skill that I learned during volunteering*. (Student 4 from the group G)
Calculating and dispensing medications	During the volunteer service-learning experience, the students reported that they had the opportunity to learn drug calculations and dispense medications to the patients	I progressed from first learning to interpret prescriptions to dispense medications, and finally to compounding medications under the guidance of the pharmacist (Student 3 from Group H)
Taking drug inventory	Part of the nursing process involves observation, planning, implementation, and evaluation. Nurses are required to maintain a proper inventory of medications and items they use in the ward for their patients. Although the setting for the volunteer was pharmacy-based, students reported learning to take an inventory of medications.	*In addition to checking expiration dates*, *I also learned to take inventory of the drugs*. *I only performed this task when asked by the pharmacist*. *(Student 6 from Group I)*
Taking patients vital signs	One fundamental responsibility of nursing care is the accurate measurement of vital signs. Most pharmacy outlets in Belize measure blood glucose levels and hypertension in patients. Many students reported learning to measure blood glucose levels and blood pressure during volunteer service.	*The pharmacy where I volunteered test patients for diabetes and checked their blood pressure*. *As soon as I got there*, *I was taught to perform these measurements and it became part of my daily routine*.* *.* *.* *. *I can now check blood glucose and BP accurately and with confidence*. (Student 1 from Group I)
Patient education	The students reported that they could counsel the patients about medication dosages, frequency of medication intake, and other useful information about the medication. A few students reported that they could discuss the etiology and pathophysiology of some diseases with customers.	*I learned to do patient counseling when I dispense medication to the patient*. *I was told that even if the customer did not ask for information on the medication*, *such as side effects*, *I should tell them*, *so they are made aware…* (Student 3 from Group B)
Communication skills in healthcare	Students reported gaining new communication skills or improved their communication skills as they interacted with the pharmacist and customers.	*……*. *even though I am somehow shy*, *I made deliberate efforts to improve my communication with the pharmacist and also with the customers*. *I watched and listened when the pharmacist discussed with the patient*. *Overall*, *the experience helped build my confidence to listen and be more expressive*. (Student 5 from Group A)

### 4. Inter-professional relationship in healthcare

In addition to interacting with patients, nurses regularly interact with other healthcare professionals in the line of duty. The students reported that the volunteer provided them with an opportunity to interact with other healthcare professionals, especially pharmacists and pharmacy assistants.

*This entire experience was an eye-opener from the start to the end*. *I felt like I grew as much as a person*, *nurse*, *and patient educator*. *Even though I had previous experience in the hospital setting*, *I think it is a good idea to get a little insight into other professions related to yours*. *I noticed that we get to learn much more and understand things better when we are placed in different environments*, *but somehow still related to our field*. (Student 2 from Group C)

### 5. Challenges and suggestions to pharmacy service-learning

Most of the students reported that volunteer experience was a helpful experience, even though several of them experienced a few challenges. The most common challenge reported by the participants was anxiety due to lack of experience. Other students reported that they were not sure how the preceptors would respond to them because they were nursing students. A few mentioned challenges such as how to manage school work and volunteer service, and whether they will be able to communicate effectively or learn the medications at the pharmacy. Some of the quotes expressed students’ views:

*First I was nervous*. *Then*, *I was also unfamiliar with all the drugs and their presentations*. *Sometimes I felt a bit embarrassed about not being familiar with a certain drug*, *so I would go in the pharmacy when am free and search for the drugs I was not familiar with in order for me to familiarize myself with the various unfamiliar drugs*. *Eventually*, *the pharmacist was really nice*, *made me comfortable*, *and took time to teach me the drugs*. *I ended up liking my experiences*. (Student 1 from group H)

The students also recommended that the experience should be ongoing.

*My friend who took this course last year told me it was very challenging*, *so I enrolled with some anxiety*. *However*, *with the pharmacy volunteer*, *and my interactions with the pharmacists and the teachers*, *I had a great experience*. *I will recommend this to be ongoing for future students*. *It was a rewarding experience for me*. *(Student 1 from Group C)*.

## Discussion

The second-year baccalaureate nursing students were assigned 48-hours voluntary community/hospital pharmacy service-learning to help familiarize them with different medications used in clinical practice. Deficiencies in nursing students’ pharmacological knowledge bases have been previously reported [28–30]. Iranian nursing students have reported the challenge of effectively teaching pharmacology and medication management as the weakest performance of nursing pharmacology instructors [31]. Although this study targeted pharmacy practice sites in contrast to hospital clinical practice sites reported by most studies, the results of the present study corroborated the studies that reported positive outcomes as a result of exposing students’ nurses to practical clinical experiences [32–34]. However, the results of this study are in contrast to the research by Sharif and Masoumi [35], who reported that nursing students in Iran were not satisfied with the clinical component of their training.

Results from the students’ reflective journals and focus group interviews supported each other and showed that the students learned the different classes of drugs, their indications, adverse effects, pregnancy categories, and contraindications of the medications they encountered at the pharmacy, an indication of the effectiveness of the teaching approach. Recent reviews enumerated learning strategies for training nurses to include simulation, technology, collaborative learning, peer learning, and research-based strategies [2,36,37]. The success of the teaching strategy employed in this study may have been a result of motivation and interest stimulated by the lecturers and the desire for students to explore new learning methods. Participation in extracurricular activities, students’ interests, and motivation have been reported as student-related factors that could play essential roles in undergraduate nursing students’ academic engagement [38]. The findings of this study further support the need for nursing pharmacology instructors to explore unconventional strategies such as pharmacy service-learning in engaging students with activities that will facilitate the learning of medications.

In addition to learning medications, most nursing students reported that they learned a few skills that are useful to medication safety and the overall nursing profession (Table 2). This study corroborated some reports in which students learned skills during clinical posting [39,40]. For instance, healthcare workers’ communication is an essential tool that fosters interactions and satisfaction between healthcare personnel and patients. In nursing, communication is a crucial tool for the interaction between the healthcare team, patients, and all aspects of nursing management [41,42]. Therefore, learning practical communication skills is relevant to nursing students because ineffective communication can lead to errors in diagnoses, anxiety, lack of patient satisfaction, and poor treatment outcomes. Ineffective communication among healthcare teams has been reported as a common cause of medical errors [42,43]. Training that will improve communication among nursing students is therefore significant in reducing therapeutic errors [43].

The opportunity to learn to interpret prescriptions and dispense medications was another notable finding of this study. Accurate interpretation of prescriptions, dispensing medications, and administering medications are vital roles of nurses that ensure their patients’ safety. Therefore, proper medication safety is critical in preventing medication errors, and nursing students must have mastery of this skill for better therapeutic outcomes [44]. In Indonesia, students reported a lack of knowledge, skills, supervision, and good role models as significant causes of medication errors [44]. Therefore, this study indicated that nursing students had the opportunity to gain skills in prescription interpretation and dispensing, which play vital roles in medication safety.

Another notable skill reported by the students was their ability to learn the basics of patient education. One key role of nurses is patient education. This dynamic skill starts from admission and continues throughout therapy until the patient is discharged. Effective patient education helps reduce the incidence of complications, improve patient quality of life and satisfaction, increase patient participation in healthcare activities, and reinforce positive patient behaviors while reducing admission rate and cost [39,45]. Poor quality of education and communication skills have been attributed to unsatisfactory patient education, leading to patients’ perception that good counseling or information was not provided [39,46]. A recent study in Iran reported barriers to patient education, including lack of self-confidence, lack of willingness and motivation, and insufficient academic knowledge expression in a simple way for the patient to understand [47]. Although this study was not conducted in a clinical setting, nursing students’ opportunities to interact with patients and provide primary patient education were considered a crucial input to their learning. Confidence in nursing students will support classroom learning and a foundation for future interaction with their patients, making the initiative a beneficial experience.

Learning to work with preceptor pharmacists as members of the healthcare team was reported by most nursing students in this study. In previous studies, student nurses reported learning from registered nurses and other professionals during ward round discussions or observations [37,48,49]. Wang et al. [50] assessed nurse-pharmacy collaboration in Wuhan, China, and reported positive attitudes toward nurse-pharmacist collaboration. With emerging global health problems such as COVID-19 and related diseases, health professionals need to work as teams to tackle global crises effectively. Teamwork among health professionals can be achieved by a clear definition of responsibilities and roles, respect and trust, effective open communication lines, and the establishment of common goals [51,52]. Moreover, when healthcare professionals work as a team, there is an improvement in the implementation of responsibilities and competencies, integration of values, accountability, and synergies [52]. Personal attributes, willingness, and professionalism were some of the characteristics reported by clinical supervisors that were important for students’ preparedness for clinical learning [53]. The report by nursing students on learning to work as members of the healthcare team is consequently significant. Belize as a country is multicultural and multi-ethnic; as such, nursing students’ opportunity to work with diverse healthcare professionals at the pharmacy practice sites was an advantage to the students since it was introduced early in their training.

Finally, even though most students reported positive responses to the service-learning experiences, a few students reported challenges they faced either before or during the experience. Anxiety is the most reported challenge among students. Nursing students’ challenges during clinical practice have been reported in several studies [34,54,55]. In a recent review, Panda et al. [55] reported that the attitudes of instructors, clinical staff, and significant others have substantial influences on students’ learning during clinical postings. The perceived fear of making errors, lack of self-motivation, and a lack of a sense of belonging, theory and practice inconsistencies, and workload were some of the daunting aspects among nursing students during clinical practice [55]. The challenges reported in this study were sufficiently overcome by the students to achieve the objectives of the volunteer service. The students were highly motivated to learn hence, the challenges did not become daunting. In addition, the lecturers and preceptors provided support and commitment to the students to assuage their fears and anxieties. Overall, the volunteer experience provided opportunities for second-year baccalaureate nursing students to integrate theory with practice, learn skills, and work as part of the healthcare team.

Therefore, the present study’s findings are significant because they suggested that combining classroom instructions with pharmacy experiential learning effectively complements teaching nursing pharmacology. The service-learning provided the students the opportunity to learn pharmacologic knowledge through direct interactions with medications, pharmacists, and lecturers and to apply introspection to reflect on the pharmacologic learning experience, thereby bridging the gap between conventional classroom instruction with experiential service-learning [21]. Because pharmacology is a challenging subject for many nursing students, applying Kolb’s [21] experiential theory of learning in this study positively facilitated learning through service-learning and reflective journals. Based on Kolb’s [21] experiential learning theory, the results of this study indicated that nursing students passed through a concrete learning experience, reflective observation, abstract conceptualization, and active experimentation to learn pharmacologic knowledge during the pharmacy volunteer service. The availability and accessibility of training facilities, inadequate training for preceptors, students’ perceptions and preparedness, and finances could be barriers to implementing this teaching strategy. Effective planning and training could help implement innovative teaching methods reported in this study.

## Conclusions

This study highlights the importance of experiential learning of pharmacology amongst second year nursing students, offering the opportunity to inform and support pharmacotherapeutics educators in designing strategies for more effective teaching of medications to nursing students. It also supports the addition of pharmacy placements to the nursing curriculum’ as it shows that nursing students can learn medications, skills, and teamwork from experiential pharmacy site posting. Combining classroom instruction with pharmacy experiential service-learning might be an effective complement for teaching nursing pharmacology.

### Limitations

The present study has some limitations, and the results should be interpreted with caution. Although two qualitative data collection approaches were utilized, the results were still self-reported, and the possibility of recall bias may not be ruled out. The study did not compare different teaching strategies to assess the best approach for a unique learning environment. The qualitative data generated were extensive due to the large number of students in the study, and not all analysed data were presented. Furthermore, caution needs to be exercised in utilizing this teaching approach to not confuse the students in their training to become professional nurses, primarily as this study was only conducted in one institution and only in the country of Belize. The study needs to be replicated in different settings using a larger sample size to ensure generalizability. Finally, although the students reported positive experiences, possible bias could have been introduced during the interviews as the students may be tempted to report what the instructors want to hear. Despite these limitations, this study is unique. The methodological approaches and the fact that this study has not been conducted in Belize, the Caribbean, and the entire Central America, make the study unique. It provides baseline data for the design and further exploration of this teaching strategy, overall, giving the study valuable and encouraging strengths.

## Supporting information

S1 File Tool focus group interview guide. (DOCX)
