# Supplementary material for: Nursing students’ experiences of service-learning at community and hospital pharmacies in Belize: Pedagogical implications for nursing pharmacology
Source: PLoS One. 2022 Nov 3;17(11):e0276656. doi: 10.1371/journal.pone.0276656 (PMC9632813; doi:10.1371/journal.pone.0276656)
Supplement: S1 File — (DOCX) [file pone.0276656.s001.docx]

**S1 Tools. Focus group interview guide**

**Question:** Share with us your experience during the pharmacy volunteer service.

*Follow up questions:*

If you get the chance to practice nursing, do you think you have gained adequate pharmacotherapeutic (medication) knowledge and confidence from the volunteer service experience?

What did you learn about medications during your service-learning experience?

Tell us the three skills you have learned during your service-learning in the community/hospital-based pharmacy and why do you think those skills are critical to medication safety in nursing practice?

We have heard students saying that most service learning is challenging. What do you think*?*

In your opinion, what would be the best solution for eliminating the challenges of learning pharmacotherapeutics in the nursing course?

Do you find your experience in service-learning pedagogically rewarding to the nursing course in pharmacotherapeutics?

Do you think Service-learning in a community/hospital-based pharmacy is better than traditional lectures in the classroom?

*Concluding question*

Is there anything else you would like to share regarding your pharmacy service-learning experience?
